# Interpain A, a Cysteine Proteinase from *Prevotella intermedia*, Inhibits Complement by Degrading Complement Factor C3

**DOI:** 10.1371/journal.ppat.1000316

**Published:** 2009-02-27

**Authors:** Michal Potempa, Jan Potempa, Tomasz Kantyka, Ky-Anh Nguyen, Katarzyna Wawrzonek, Surya P. Manandhar, Katarzyna Popadiak, Kristian Riesbeck, Sigrun Eick, Anna M. Blom

**Affiliations:** 1 Lund University, Department of Laboratory Medicine, Section of Medical Protein Chemistry, University Hospital Malmö, Malmö, Sweden; 2 Jagiellonian University, Department of Microbiology, Krakow, Poland; 3 University of Georgia, Department of Biochemistry and Molecular Biology, Athens, Georgia, United States of America; 4 Westmead Millennium Institute, Institute of Dental Research, Sydney, Australia; 5 Lund University, Department of Laboratory Medicine, Section of Medical Microbiology, University Hospital Malmö, Malmö, Sweden; 6 Department of Medical Microbiology, University Hospital of Jena, Jena, Germany; Umeå University, Sweden

## Abstract

Periodontitis is an inflammatory disease of the supporting structures of the teeth caused by, among other pathogens, *Prevotella intermedia*. Many strains of *P. intermedia* are resistant to killing by the human complement system, which is present at up to 70% of serum concentration in gingival crevicular fluid. Incubation of human serum with recombinant cysteine protease of *P. intermedia* (interpain A) resulted in a drastic decrease in bactericidal activity of the serum. Furthermore, a clinical strain 59 expressing interpain A was more serum-resistant than another clinical strain 57, which did not express interpain A, as determined by Western blotting. Moreover, in the presence of the cysteine protease inhibitor E64, the killing of strain 59 by human serum was enhanced. Importantly, we found that the majority of *P. intermedia* strains isolated from chronic and aggressive periodontitis carry and express the interpain A gene. The protective effect of interpain A against serum bactericidal activity was found to be attributable to its ability to inhibit all three complement pathways through the efficient degradation of the α-chain of C3—the major complement factor common to all three pathways. *P. intermedia* has been known to co-aggregate with *P. gingivalis*, which produce gingipains to efficiently degrade complement factors. Here, interpain A was found to have a synergistic effect with gingipains on complement degradation. In addition, interpain A was able to activate the C1 complex in serum, causing deposition of C1q on inert and bacterial surfaces, which may be important at initial stages of infection when local inflammatory reaction may be beneficial for a pathogen. Taken together, the newly characterized interpain A proteinase appears to be an important virulence factor of *P. intermedia*.

## Author Summary

*Prevotella intermedia* is one of the bacterial pathogens that has been implicated in causing periodontitis—an endemic inflammatory disease of the supporting structures of the teeth. The complement system is an important part of host innate immunity and is able to directly kill invading bacteria. To become successful pathogens, many strains of *P. intermedia* developed mechanisms making them very resistant to killing by complement. We found that a cysteine protease, interpain A, that is produced by many clinical strains of *P. intermedia* was able to destroy the bacterial killing activity of human serum. A strain of *P. intermedia* that produces interpain A was found to be more resistant to complement than the one lacking interpain A, and the resistance of the interpain A–producing strain could be diminished by a specific inhibitor of cysteine proteases. We attributed the protective effect of interpain A to its ability to inhibit the complement system through the efficient degradation of C3—a major complement protein that is common to all three pathways of complement activation. Understanding the mechanism governing pathogen resistance to complement may help us to design novel therapeutic strategies to prevent or treat an important bacterial disease.

## Introduction

Periodontitis is an inflammatory condition with an infective etiology that leads to loss of tooth support. *Prevotella intermedia* is a major bacterial periodontal pathogen in humans together with *Porphyromonas gingivalis* and *Aggregatibacter actinomycetemcomitans* [1]. *P. intermedia* is often recovered from subgingival plaque in patients suffering from acute necrotising gingivitis, pregnancy gingivitis and chronic periodontitis [2]. Recently, *P. intermedia* was reported to be found in 14% of adult population in Finland and there was association between the carriage of this species and the number of teeth with deepened periodontal pockets [3]. *P. intermedia* was also frequently isolated from root canal infections [4]. Periodontitis is one of the most common diseases affecting humans and is primarily the result of colonization of the subgingival surfaces of teeth by bacteria. The complex interaction between these bacteria harboring many virulence factors and the host's immune response results in localized chronic inflammation and subsequent destruction of the supporting structures of the tooth. Proteinases are crucial virulence factors produced by many periodontal pathogens, which can cause the degradation of host proteins for essential nutrients but they can also protect the bacteria from the host's defenses such as the complement system [5],[6].

Complement is a major arm of the innate immune defense system and its main function is to recognize and destroy microorganisms [7]. The three pathways of human complement ensure that virtually any non-host surface is recognized as hostile. The classical pathway is usually mediated by binding of the C1 complex (composed of recognition molecule C1q and two proteinases C1s and C1r) to invading pathogens either directly or via immunoglobulins. The lectin pathway is able to recognize, via mannose-binding lectin (MBL), polysaccharide molecules normally present only on microbial surfaces. Finally, complement can also be activated through the alternative pathway, which is not so much an activation pathway but as a failure to appropriately regulate the constant low-level spontaneous activation of C3 (constantly initiated due to inherent instability of this protein). All three pathways lead to opsonisation of the pathogen with C3b (activated form of complement factor C3), which enhances phagocytosis by phagocytes. Furthermore, anaphylatoxins C5a and C3a are released as byproducts to attract phagocytes to the site of infection. Finally, the end result of the complement cascade is formation of the membrane attack complex and bacterial cell lysis. Host cells protect themselves from bystander damage following complement activation through the expression of membrane-bound or recruitment of soluble endogenous complement inhibitors.

Complement deficiencies are very rare but it has been observed that partial C4 gene deficiencies are more frequent in patients with severe chronic periodontitis [8]. A patient with aggressive periodontitis and severe edema, localized to the free gingival tissues was reported to be deficient in C1-inhibitor [9]. Furthermore, the highest salivary levels of C3 were measured in periodontally healthy subjects while low levels were often found in edentulous and chronic periodontitis patients [10].

It has been demonstrated that heat inactivation of NHS (i.e. inactivation of complement) significantly reduced opsonic activity for *P. intermedia in vitro* [11] suggesting that complement is important for host defense against this pathogen. Previous studies have shown that *P. intermedia* was opsonized by the alternative pathway in the absence of the classical pathway, probably in response to the endotoxin [12], however, kinetic studies revealed that opsonisation proceeded at significantly faster rates when the classical pathway was intact [11]. Interestingly, the alternative pathway contributed to the killing of serum sensitive strains while the classical pathway was primarily responsible for killing of strains with intermediate sensitivity [13]. Therefore, it appears that complement is able to recognize *P. intermedia* via several sensory molecules. However, it appears that *P. intermedia* is able to override to some extent the complement defenses and to establish chronic infections in the oral cavity.

Every successful human pathogen must develop means to circumvent complement. Many bacteria are able to capture human complement inhibitors such as C4b-binding protein and factor H thereby inhibiting complement and avoiding opsonisation and lysis [14]–[16]. Herpes viruses, on the other hand, produce their own homologues of complement inhibitors [17]. Furthermore, many bacteria use proteinases to incapacitate components of the complement system. For example, most strains of *P. gingivalis* are resistant to bacteriolytic activity of human serum [13],[18] and the gingipain proteinases have been implicated as the major factor providing protection against complement in serum [5], [19]–[22]. For a number of *Prevotella* subspecies and strains, including *P. intermedia*, the level of proteolytic activity for clinical strains was significantly higher than that recorded for commensal strains isolated from healthy mouths [23]. This, we hypothesize, may provide *P. intermedia* with serum protection.

We have identified three cysteine proteinases in the genome of *P. intermedia* that appeared to be homologues of SpeB protein of *Streptococcus pyogenes* [24]. Recently, the first of these genes coding for interpain A (InpA; locus PIN0048) was studied in more detail and its 3D structure was determined [25]. Based on similarity of primary and tertiary structures to known proteinases, InpA is now classified into clan CA, family C10 and registered in the peptidase database MEROPS ([26]; http://merops.sanger.ac.uk). InpA is a secreted protein composed of 868 amino acid residues including a 44-residue signal peptide, a pro-domain (Ala1-Asn111), a catalytic domain (Val112-Pro359) and a 465-residue C-terminal extension arranged in domains with putative regulatory and secretory functions. However, the specific target(s) and function of InpA have yet to be characterized. In the present study, we have examined in detail the effect of InpA on the human complement system and found that this proteinase targets mainly the C3 component, thereby inhibiting all three complement pathways simultaneously.

## Results

### *inpA* gene is present in the majority of the clinical isolates of *P. intermedia*

**Figure 1:**
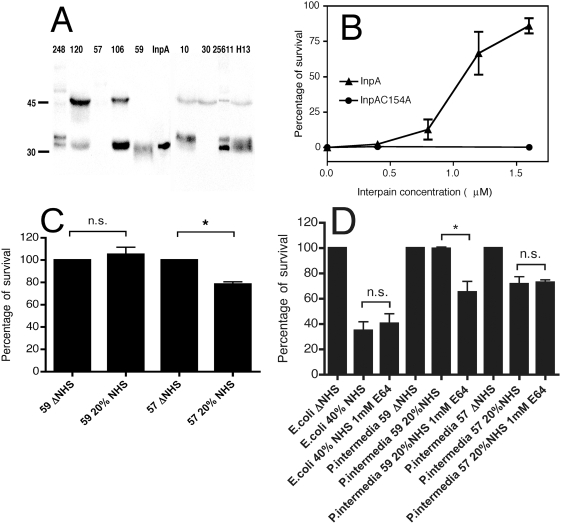
Interpain A destroys bactericidal activity of NHS. (A) Western blotting analysis of InpA expression. Five-day old broth cultures of nine *P. intermedia* strains were adjusted to OD_600_ of 2, and the culture supernatants were separated by SDS-PAGE under reducing conditions and proteins transferred onto PVDF membranes. InpA was visualized with polyclonal antibodies. One lane shows purified InpA. (B) Western blotting analysis of gingival crevicular fluid (20 µL/lane) from patients with chronic periodontitis. Load of *P. intermedia* was determined with qPCR (<100 bacteria/sample “−”, 100–1,000 bacteria/sample “+”, 10,000–50 000 bacteria/sample “++”, >50,000 bacteria/sample “+++”). The leftmost lane contains 1 µg of a purified inactive recombinant mutant, InpAC154A. (C) *E. coli* DH5α were incubated with 2% NHS pretreated with increasing concentrations of InpA and InpAC154A, and the surviving bacteria were enumerated after overnight culture on LB agar plates. As a control, heat-inactivated NHS (ΔNHS) was used, and the survival of bacteria in this condition was set to 100%. (D) *P. intermedia* and *E. coli* were incubated with 20% NHS and 40% NHS for 1.5 h in anaerobic conditions, respectively, with and without supplementation with 1 mM E64 protease inhibitor, and the surviving bacteria were enumerated after culture onto TSB and LB plates, respectively. In (C) and (D) an average of three independent experiments is presented with bars indicating standard deviation (SD). Statistical significance of observed differences was estimated using Student's t-test; n.s. not significant, *p<0.05.

In order to estimate what fraction of *P. intermedia* strains found in periodontitis carry the *inpA* gene, detection by PCR was used on subgingival plaque samples obtained from 24 and 58 patients with chronic and aggressive periodontitis, respectively. We have validated specificity of the PCR assay by investigating 25 samples that were negative for *P. intermedia* but rich in other periodontal pathogens. No positive signal was obtained in any of the tested *Prevotella*-negative samples showing that the assay is specific for *Prevotella inpA* gene (data not shown). *P. intermedia* was detected in 33% and 57% of plaque samples from chronic and aggressive periodontitis, respectively (Table 1). The majority of *P. intermedia-*positive samples also yielded positive results regarding the *inpA* gene implying that InpA fulfils some important physiological function. Similarly, we found that the majority of cultivated *P. intermedia* strains from various sources also express InpA at the protein level as shown by Western blotting analysis of culture supernatants (Figure 1A). The upper band recognized by the specific antibody corresponds to an unprocessed form of InpA while the lower bands are products of autocatalytic processing [25]. Western blotting of lysates of bacterial cells did not yield a signal implying that InpA is mainly secreted by the bacteria and does not associate in large amounts with cell wall in the strains tested (data not shown).

**Table 1:** Prevalence of *Prevotella intermedia* and the presence of interpain A in subgingival plaque samples.

Group (Numbers)	Prevalence	High Load (≥10^6^)	*inpA* Gene Present
Healthy controls	0	0	
Aggressive periodontitis (24)	8 (33%)	3 (13%)	6 of 8 (75%)
Chronic periodontitis (58)	33 (57%)	22 (38%)	25 of 31 (81%)

Furthermore, we have detected InpA protein in gingival crevicular fluid samples collected from four chronic periodontitis patients characterized with regard to pocket depth and bleeding-on-probing. The samples were analyzed for the *P. intermedia* load using qPCR and subjected to Western blotting analysis. We detected InpA in various forms in samples obtained from patients with significant load of *P. intermedia* but not from those negative for this pathogen (Figure 1B). The 90 kDa form is the unprocessed full length protein, the 76 kDa and the 40 kDa proteins are processed on the N-terminus and the C-terminus, respectively, while the 28 kDa form is the mature, fully-processed protein. These molecular weights are calculated based on amino acid composition. The 40 kDa form runs in fact as 45 kDa protein (28 kDa form as 32 kDa protein) upon separation on 12% SDS-PAGE gel.

### Interpain A destroys the bactericidal activity of human serum

In order to quantitatively assess the effect of purified InpA on the bactericidal activity of human serum, we used an *E. coli* DH5α model system whereby cells were incubated with normal human serum (NHS) pretreated with various concentrations of InpA or its inactive mutant (InpAC154A) and surviving cells enumerated by colony counting. InpA was found to be able to destroy the bactericidal activity of human serum in a dose-dependent manner and rescued *E. coli* that are otherwise very sensitive to killing by NHS (Figure 1C). Moreover, *P. intermedia* strains have been known to vary significantly in their ability to resist killing by NHS [13], hence, various strains were investigated to see if there was a relationship between the serum resistance of a given strain and its InpA expression level. By Western blotting, *P. intermedia* strain 59 producing a large amount of InpA was found to have a 100% survival rate in 20% NHS while only 78% of the strain 57 with non-detectable InpA production survived (Figure 1A and 1D). Furthermore, addition of a cysteine proteinase inhibitor E64 to NHS decreased the ability of *P. intermedia* strain 59 to survive while it did not affect the killing of strain 57 or *E. coli* (Figure 1D). Taken together, the results obtained with both purified InpA and *P. intermedia* strains showed that InpA compromised the bactericidal activity of human serum.

### Interpain A destroys complement system in human serum

**Figure 2:**
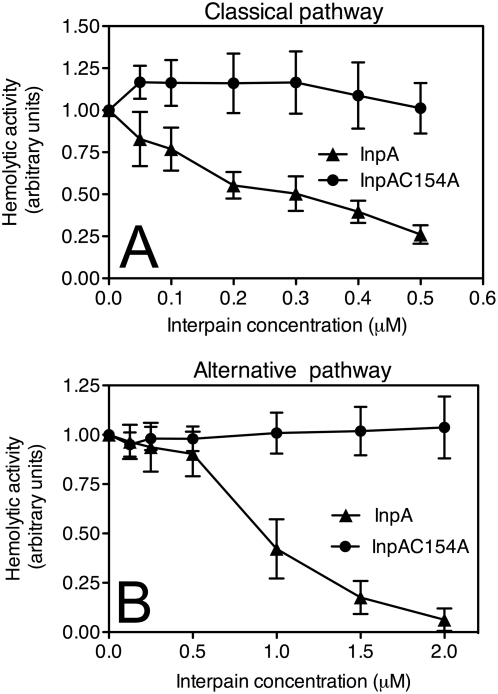
Interpain A destroys hemolytic activity of human serum. Sheep erythrocytes sensitized with antibodies (classical pathway, (A)) or rabbit erythrocytes (alternative pathway, (B)) were incubated with 1.25% or 10% NHS, respectively. Serum was supplemented with various concentrations of InpA and InpAC154A. After 1 h incubation, the degree of lysis was estimated by measurement of released hemoglobin (absorbance at 405 nm). Lysis obtained in the absence of interpain was set as 1. An average of three independent experiments is presented with bars indicating SD.

In order to understand in detail how InpA destroys the bactericidal activity of NHS, i.e. complement, the enzyme was incubated at various concentrations with human serum and hemolytic assays were used to assess activity of the classical and alternative pathways of complement in the pre-treated sera. InpA was found to be an efficient inhibitor of both pathways, whereas the inactive mutant InpAC154A did not show any inhibition (Figure 2A and 2B). InpA was able to inhibit the classical pathway by 80% when present at high nanomolar concentrations (0.5 µM) while the alternative pathway was inhibited by 80% at 1.5 µM concentration. It should be noted, however, that 10% serum was used for the alternative pathway hemolytic assay versus 1.25% for the classical pathway. These concentrations were chosen on a basis of the initial titration and represent conditions in which each assay was most sensitive. The alternative pathway is known to require high concentrations of serum in order to function properly in contrast to the classical pathway that is rapidly activated even at fractions of percent of NHS. Taken together, it appears that InpA is approximately equally able to destroy activity of both the classical and alternative pathways.

### Interpain A interferes with all three pathways by degrading mainly C3

**Figure 3:**
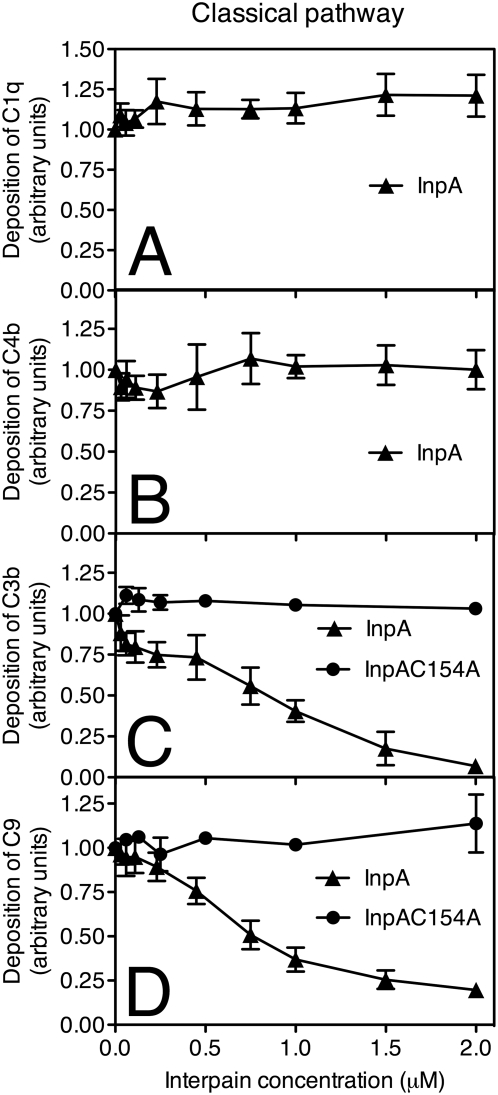
Interpain A inhibits the classical pathway. InpA and InpAC154A were incubated with 2% NHS (for C1q, C4b, C3b assays) and 10% NHS (for C9 assay) for 15 min and inactivated with an excess of E64 (20 µM) to prevent degradation of IgGs. Human IgGs were immobilized on microtiter plates and allowed to activate NHS pre-incubated with various concentrations of InpA and InpAC154A. After 20 min (C3b, C4b) and 45 min (C1q, C9) of incubation, the plates were washed, and deposited C1q (A), C4b (B), C3b (C), and C9 (D) were detected with specific polyclonal antibodies. Absorbance obtained in the absence of InpA was set as 1.0 unit. An average of three independent experiments is presented with bars indicating SD. Data points without error bars have minimal SD, which are not displayed by the graphing software (GraphPad Prism 4).

Each complement pathway is composed of several factors activated in a consecutive manner. In order to assess which complement factor(s) were affected by InpA, a microtiter plate-based assay in which complement activation was initiated by various ligands depending on the pathway analyzed was used and the deposition of successive complement factors was then detected with specific antibodies. In the case of the classical pathway, complement activation was initiated by immunoglobulin deposition. We found that depositions of C1 and C4 from 2% serum were not affected by InpA (Figure 3A and 3B). However, C3 was found to be sensitive to InpA and deposition of C3b from NHS was abolished at 2 µM InpA (Figure 3C). The inactive InpAC154A mutant had no effect on activation and deposition of C3b at any concentration tested. Accordingly, deposition of C9 that appears in the cascade after C3 was inhibited at similar concentrations as C3 indicating that the inhibitory effect on deposition of C9 was due to degradation of C3 (Figure 3D).

**Figure 4 ppat-1000316-g004:**
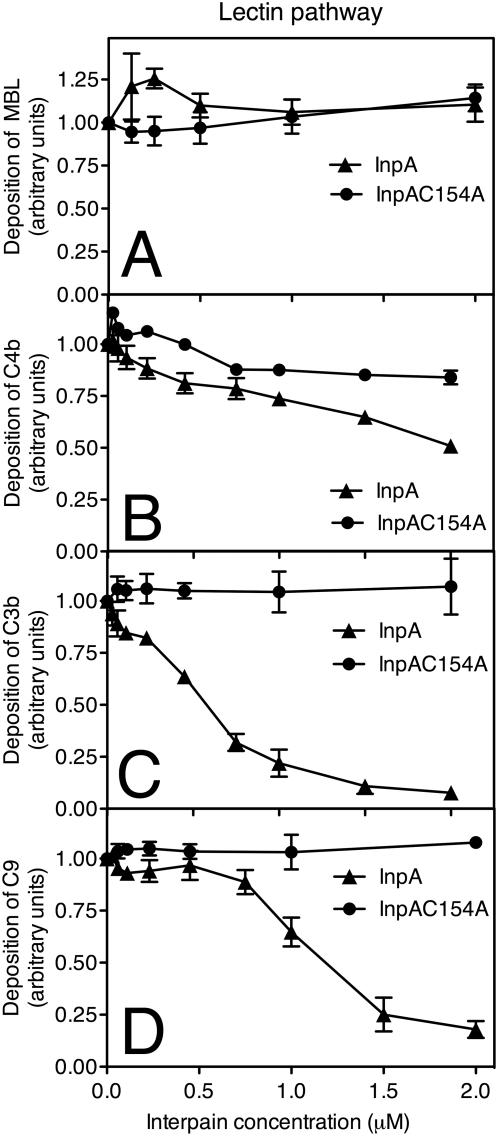
Interpain A inhibits the lectin pathway. Mannan was immobilized on microtiter plates and allowed to activate 4% NHS (for MBL, C4b, C3b assays) and 10% NHS (for C9 assay) that was pre-incubated for 15 min with various concentrations of InpA and InpAC154A. After 20 min (C3b, C4b) and 45 min (C9, MBL) of incubation, the plates were washed, and deposited MBL (A), C4b (B), C3b (C), and C9 (D) were detected with specific polyclonal antibodies. Absorbance obtained in the absence of interpain was set as 1. An average of three independent experiments is presented with bars indicating SD.

For assessment of the lectin pathway, we used plates bound with mannan carbohydrate. In this case, InpA did not affect the binding of MBL, which is the initiator of the pathway (Figure 4A) and weakly inhibited deposition of C4b (Figure 4B). However, similar to the classical pathway, InpA strongly inhibited the deposition of C3b and C9 while the InpAC154A mutant had no effect (Figure 4C and 4D).

**Figure 5:**
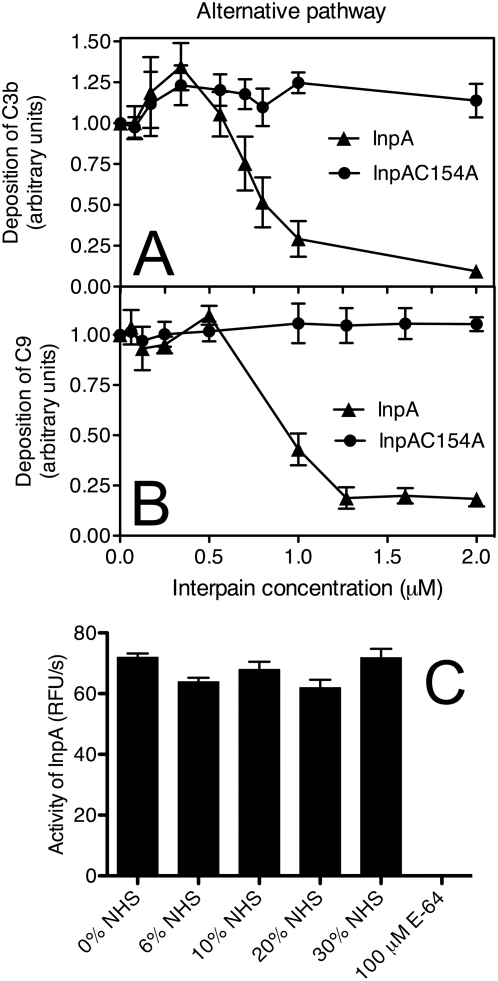
Interpain A inhibits the alternative pathway. Zymosan was immobilized on microtiter plates and allowed to activate 6% NHS (for C3b assay) and 10% NHS (for C9 assay) that was pre-incubated for 15 min with various concentrations of InpA and InpAC154A. After 20 min (C3b) and 45 min (C9) of incubation, the plates were washed, and deposited C3b (A) and C9 (B) were detected with specific polyclonal antibodies. Absorbance obtained in the absence of interpain was set as 1. (C) InpA was incubated with increasing concentrations of NHS, and the activity of InpA was determined using a synthetic substrate. The cysteine protease inhibitor E64 was used as a control. An average of three independent experiments is presented with bars indicating SD.

The alternative pathway was activated by immobilized zymosan and InpA was found to be able to inhibit deposition of C3b and C9 with a similar efficiency as previously found for the other two pathways (Figure 5A and 5B). Taken together, all three pathways were sensitive to InpA and its main target appeared to be C3, which is the key protein for all pathways of the complement system.

NHS contains several proteinase inhibitors that could potentially inhibit the activity of InpA. However, we found that the InpA activity measured with fluorogenic substrate was not affected by NHS when NHS was present at concentrations up to 30% (Figure 5C).

### Interpain A attacks preferentially α-chains of C3 and C4

**Figure 6:**
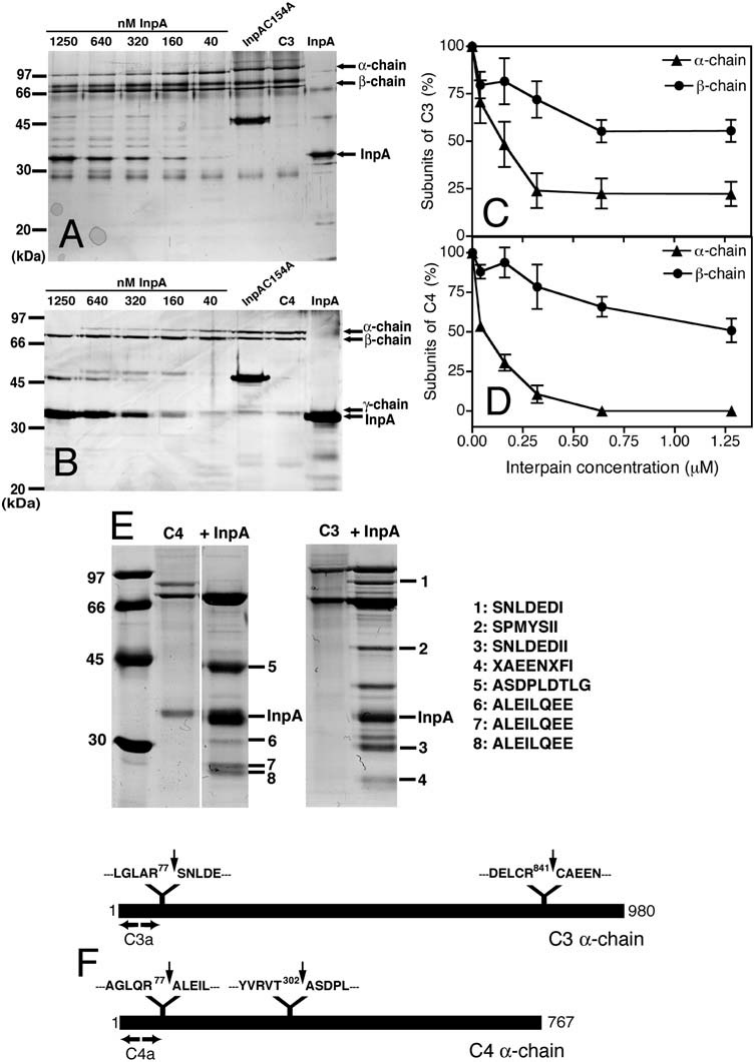
Interpain A degrades preferentially α-chains of C3 and C4. Purified C3 (A,C) and C4 (B,D) were incubated for 30 min with increasing concentrations of InpA or 1250 nM of InpAC154A and separated by SDS-PAGE. The gels were stained with silver (A,B), and protein band intensities corresponding to the α-chains and β-chains of C3 and C4 were analyzed by densitometry (C,D). The graphs show the % of native α- and β-chains remaining after incubation with InpA. An average of three independent experiments is presented with bars indicating SD. (E) In order to determine cleavage sites in C3 and C4, these proteins were digested and subjected to N-terminal sequencing. The N-terminal sequences of selected bands are listed on the right. (F) A schematic representation of C3 and C4 α-chains with indicated sites of cleavage by InpA.

In order to assess the sites cleaved by InpA, in complement factors, purified C3 and structurally related C4 were incubated with InpA at various molar ratios. The proteins were then separated by SDS-PAGE and visualized using silver staining (Figure 6A and 6B). C3 is composed of covalently linked α- and β-chains while C4 contains α-, β- and γ-chains. For both proteins, InpA first attacks the α-chain while the β-chain is relatively resistant (Figure 6A–6D); which is similar to what we have previously observed for gingipains [5]. The InpAC154A mutant did not cause any degradation of C3 or C4 (Figure 6A and 6B). Interestingly, similar concentrations of InpA were required for the degradation of purified C3 and C4, whereas in the presence of NHS, InpA preferentially inactivates C3 (Figures 3 and 4).

To determine sites of proteolysis by InpA, C3 and C4 were treated with InpA and degradation products were separated by SDS-PAGE electrophoresis. The proteins were transferred to PVDF membrane, visualized with Coomassie (Figure 6E) and selected bands were subjected to N-terminal sequencing. Interestingly, cleavage of the C3 polypeptide chain at the site resulting in the N-terminal sequence SNLDEDIIA generated the exact sequence of an anaphylatoxin fragment C3a. Similarly, the cleavage of C4 producing the N-terminal sequence ALEILQEE generated the exact sequence of C4a. Sequence 2 (SPMYSII) corresponds to the N-terminus of the C3 β-chain.

### Kinetic parameters of C3 and C4 degradation by interpain A

**Figure 7:**
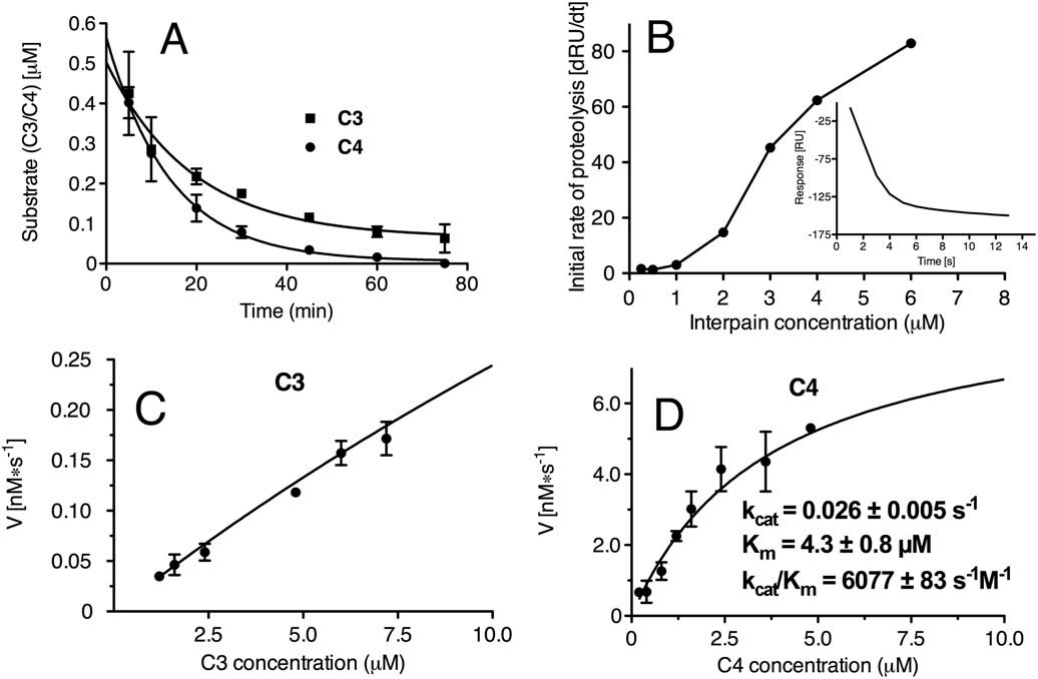
Kinetic parameters of InpA-mediated degradation of C3 and C4. (A) Degradation of C3 and C4 incubated with one concentration of InpA for different time points. (B) Kinetic measurement of degradation of C3b by InpA. Various concentrations of InpA were injected over immobilized C3b, and the reduction in RU values indicates the extent of proteolytic cleavage of C3b. The inset shows a sensorgram obtained for a 3 µM InpA sample. The initial rates of proteolysis were determined for each InpA concentration from the slope in the sensorgram and plotted as a function of InpA concentration. (C) Michaelis-Menten plot of degradation of C3 by InpA. InpA was incubated with increasing concentrations of C3, and the amount of remaining α-chains (substrate) was determined using densitometry after separation on SDS-PAGE. (D) A similar analysis as in (C) but performed for C4.

When degradation of C3 and C4 was assessed at a set concentration of InpA for increasing incubation times, C4 was degraded at a faster rate than C3 (Figure 7A). To determine the kinetic parameters of degradation of C3b by InpA, surface plasmon resonance was employed. When the inactive InpAC154A proteinase was injected over immobilized C3b, no change in signal was detected (data not shown). However, upon injection of InpA, there was a rapid decrease in the signal measured in resonance units (RU) corresponding to degradation of C3b. The initial rates of proteolysis at each concentration of InpA were obtained from the initial slopes in the sensorgrams (Figure 7B). In this system, 1000 RU corresponds to a mass shift of 1 ng/mm^2^. The analysis demonstrated that 3 µM InpA degrades C3b at an initial rate of 7 pg/s (Figure 7B inset). The kinetic parameters of C3 and C4 degradation by InpA were also determined by fitting initial rates of degradation of α-chains of C3 and C4 into Michaelis-Menten equation. A constant amount of InpA was incubated with increasing concentrations of C3 and C4 and the initial rate of proteolysis at various substrate concentrations was estimated from the decrease of intensity of scanned bands corresponding to α-chains of C3 and C4 as resolved by SDS-PAGE. Using this approach, K_m_ and k_cat_ for C4 degradation was determined to be 4.3+/−0.8 µM and 0.026+/−0.005 s^−1^, respectively (Figure 7D). Unfortunately, a reasonably accurate measurement of the kinetic constants for C3 was not possible since there was no visible saturation of the initial rate of C3 degradation up to 2 mg/mL (10 µM) of substrate, hence, the K_m_ could only be estimated as greater than 20 µM (Figure 7C).

### Interpain A causes activation and deposition of C1 in the absence of any activator

**Figure 8:**
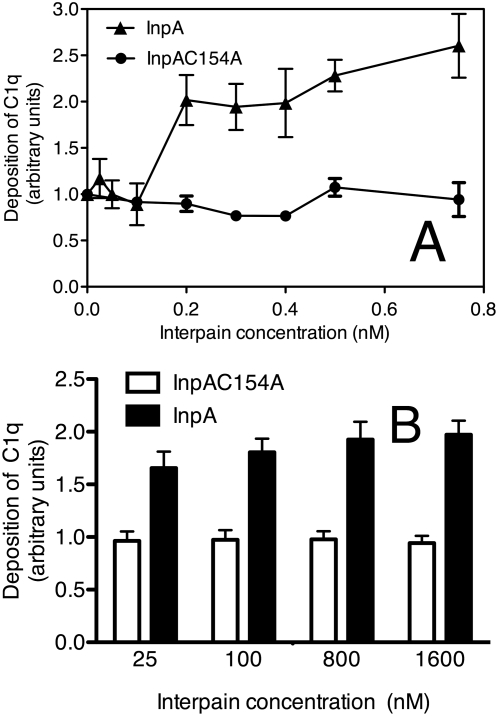
Deposition of C1q on plates and bacteria. (A) Microtiter plates were blocked with BSA and incubated with 4% NHS containing various concentrations of InpA and InpAC154A for 45 min. Deposited C1q was detected with a specific antibody, and the absorbance obtained in the absence of InpA was set as 1. (B) *P. nigrescens* ATCC 25261 was incubated with 5% NHS and different concentrations of InpA and InpAC154A. Deposition of C1q was quantified using flow cytometry with specific FITC-labeled antibodies, and the absorbance obtained in the absence of InpA was set as 1. An average of three independent experiments is presented with bars indicating SD.

We have observed previously that gingipains did not degrade C1 but instead were able to cause C1 deposition on surfaces that would not normally activate C1 [5]. In order to assess if this was also the case for InpA, human serum was incubated with InpA in the absence of any immobilized C1 activator and we observed that it did cause deposition of C1q on the empty microtiter plates blocked with BSA (Figure 8A). In the absence of InpA or in the presence of its inactive mutant, the deposition of C1q from serum was negligible as expected.

In addition, InpA was also found to be able to cause deposition of C1q on bacterial surfaces. To this end, *Prevotella nigrescens* was incubated with NHS containing InpA at different concentrations and the deposition of C1q was measured using flow cytometry. We found that addition of InpA to NHS caused an increase in deposition of C1q on the surface of *Prevotella* that mimicked results obtained using microtiter plates (Figure 8B). Taken together, our results show that InpA is able to cause deposition of active C1 complex on normally non-activating surfaces such as BSA coated plastic or bacteria. We did not observe degradation of C1q during incubation with InpA, neither when InpA was added to NHS nor when it was incubated with purified C1q (data not shown).

### Interpain A acts synergistically with gingipains

**Figure 9:**
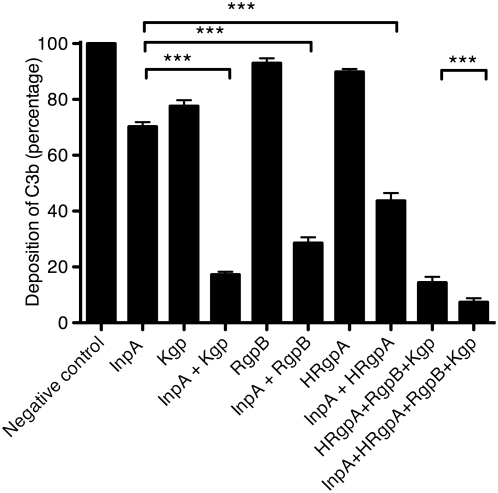
Interpain A and gingipains act synergistically. Mannan was immobilized on microtiter plates and allowed to activate 4% NHS containing 350 nM InpA and three gingipains, Kgp (44 nM), RgpB (55 nM), HRgpA (33 nM), alone or mixed together. After 20 min of incubation, the plates were washed, and deposited C3b was detected with specific antibodies. An average of three independent experiments is presented with bars indicating SD. Statistical significance of observed differences was estimated using Student's t-test; *** p<0.001.

Since InpA and gingipains are often present simultaneously at the sites of infection colonized with *P. intermedia* and *P. gingivalis*, we assessed how they acted on complement when present together. To this end, InpA and the three gingipains (HRgpA and RgpB are arginine-specific gingipains while Kgp is lysine-specific) were pre-incubated with 4% NHS at concentrations chosen to affect the activity of the lectin pathway by only 10–30%. The deposition of C3b was assessed and we found that the proteinases acted synergistically since the deposition of C3b in combinations of InpA and the gingipains was lower than predicted if the effects of the proteinases were added separately (Figure 9). For example, InpA alone decreased the deposition of C3b by 30% at the concentration used, while Kgp yielded only 25% decrease. When used together at the same concentrations, InpA and Kgp decreased C3b deposition by 85% instead of 55% that would be expected if these proteinases had only additive effects. When all three gingipains were added together with InpA, the deposition of C3b was inhibited by 93%.

## Discussion

Factors governing *P. intermedia* infection are poorly studied when compared to other periodontal pathogens such as *P. gingivalis*. However, it is becoming apparent that all successful human bacterial pathogens must develop strategies to circumvent the complement system [15]. Microorganisms in gingival sulcus are immersed in serum-derived tissue exudate—gingival crevicular fluid, which is similar in composition to human serum. Since complement components are present in gingival crevicular fluid at up to 70% of serum concentration [27] and *in vivo* there is high level of complement activation in gingival fluid of patients with periodontitis [28],[29], successful evasion of the complement system is paramount for the survival of *P. intermedia* in the periodontal pocket. One such strategy of defense against complement developed by *P. intermedia* appears to depend on the production of InpA, which we now show, is able to degrade complement factor C3, which is the central molecule of the whole complement system. Importantly, the majority of *P. intermedia* strains isolated from aggressive and chronic periodontitis carry and express the *inpA* gene.

The proteolytic activities of oral bacteria are thought to play important roles in the etiology of periodontitis and dental abscesses. These proteinases may contribute to tissue destruction, increase availability of nutrients and impair host defense by degrading immunoglobulins and components of the complement system. Proteinases of *P. intermedia* display trypsin-like and dipeptidylpeptidase activities [30] and also have the properties of cysteine proteinases [31]–[33]. They have also been reported to be capable of degrading immunoglobulins, particularly IgG [34],[35], fibronectin [36] and host proteinase inhibitors [37]. The degradation of immunoglobulins was mediated mainly by cysteine proteinase(s) [35]. Now we can add C3 to this list.

Importantly, inhibition of C3 function occurred even when InpA was incubated with whole NHS showing that C3 will be specifically degraded even in the presence of all other plasma proteins (Figures 3–5). This is not the case for C4, which was degraded efficiently when purified proteins were used but its function was only weakly affected in the presence of whole serum. According to kinetic parameters determined with purified proteins, C4 should be a far better target for InpA than C3 in serum. Despite that C4 in serum seems to be resistant to proteolytic inactivation by InpA. To explain this discrepancy, we speculate that C4 may interact with other protein(s) in serum, which hinders InpA access to a cleavage site. Alternatively, the α-chain of C4 may also be susceptible to proteolysis in serum but the cleaved protein is still a functional source of C4b. The latter explanation is supported by the observation that in contrast to C3, proteolysis of C4 is more limited (Figure 6). Such phenomenon has previously been observed for α_2_-macroglobulin, which remained functionally active after cleavage with gingipains [38]. Importantly, it is clear that InpA will affect C3 in a way that it can no longer propagate the complement cascades; which should be of direct benefit to InpA producing *P. intermedia*.

Interestingly, InpA showed a preference for the α-chain of C3 and C4, similar to what we have previously observed for gingipains. At low concentrations, gingipains were able to activate complement factors C3, C4 and C5 as they preferentially target the α-chains of these proteins to cause the release of anaphylatoxins C3a and C5a as well as the activated forms C3b, C4b and C5b. Similarly, N-terminal sequencing of C3 and C4 fragments generated by InpA revealed that InpA will also release C3a and C4a. At higher concentrations, gingipains simply degrade these three complement factors, particularly C3, into smaller fragments so that they can no longer propagate the complement cascade [19],[39]. Yet again, we observe a similar phenomenon for InpA in case of C3. Also similar to gingipains, InpA was able to cause the deposition of C1 from serum onto inert surfaces without the need for a specific C1 activator; which may lead to local inflammation. However, whereas this effect could be recreated *in vitro* using purified C1 for gingipains [5], InpA required serum to be present for this to occur (data not shown). Thus, it appears that InpA may require a third protein to induce C1 deposition from serum. Consequently, an intricate strategy emerges: periodontal bacteria at low concentrations appear to cause non-specific activation of C1 and to generate C5a and C3a fragments—chemotactic factors for neutrophils. This may lead to a low grade inflammation that provides access to nutrients for bacterial growth and colonization. At higher concentrations of bacteria and proteinases, the complement system becomes incapacitated by multiple cleavages of critical proteins within the cascade.

*P. intermedia* can be highly resistant to complement and survive at very high serum concentrations but there are significant differences between various strains with regard to sensitivity to killing by complement [13]. In this study, we have shown that there is a correlation between the presence of InpA and serum resistance of *P. intermedia*. Using *E. coli* as a sensitive model to detect bactericidal activity of human serum, we have found that they were able to survive when supplemented with low micromolar concentrations of InpA in the presence of 2% NHS. In contrast, cells exposed to NHS alone or to NHS containing the inactive interpain mutant showed total loss of viability at this serum concentration. This clearly shows that purified InpA is very efficient at destroying bactericidal activity of NHS. Further, the cysteine proteinase inhibitor E64 diminished serum resistance of *P. intermedia* strains.

It is plausible that *P. intermedia*, in similarity to other bacterial pathogens, has several strategies for evasion of killing by complement. *P. gingivalis* employs not only proteinases for defense from complement [5] but it also produces a surface anionic polysaccharide, the presence of which strongly correlates with exceptional serum resistance of these bacteria [40]. This bacterium also attenuates the effects of complement by capturing human complement inhibitor C4b-binding protein [16]. In this study, we have found that *P. intermedia* was able to retain some of its ability to resist killing even when incubated with serum containing the broad-spectrum inhibitor E64. However, InpA is a secreted protein and we do not expect large amounts of it being present in our bactericidal assay that has been performed within 1.5 h of culturing. *In vivo*, the bacteria will have the opportunity to secrete much more interpain into its pericellular environment. Our current methodology does not allow for truly quantitative analysis of the InpA content in gingival crevicular fluid. However, we can estimate from our Western blotting analysis that 20 µL of crevicular fluid contained at least 0.1 µg of InpA. Taking into account at least 20-fold dilution of crevicular fluid upon collection, the concentration of InpA in the two positive samples analyzed must be greater than 100 µg/mL. This corresponds to approximately 4 µM of fully processed InpA, implying that the concentration of InpA is high enough for inhibition of the complement system as described to occur *in vivo*.

Our experiments also showed that InpA will aid survival of bystander bacterial species, thus, creating a favorable condition for the establishment of a common ecosystem that would be a beneficial habitat for all participating species. *P. intermedia*, together with *Streptococcus gordonii* may be considered to be the early colonizers of tooth surfaces, thereby promoting secondary colonization of pathogenic organisms such as *P. gingivalis* by providing attachment sites, growth substrates and reduced oxygen concentration locally [41],[42]. *P. intermedia* belongs to the “orange complex”, which encompasses bacterial species bridging between healthy state and advanced periodontitis. Thus, degradation of C3 by InpA in synergy with gingipains of *P. gingivalis* will complement the host immune evasion strategy of subgingival microbiota. Importantly, *Prevotella* species readily acquire resistance towards antibiotics [43] and deeper knowledge of how infection and serum resistance occur will be crucial for the development of alternative treatments to periodontal disease.

## Materials and Methods

### Proteins

Purified complement proteins were purchased from Complement Technology.

InpA as well as its inactive mutant InpAC154A (the catalytic cysteine was replaced by alanine) were expressed as His-tagged recombinant proteins in *Escherichia coli* and purified by affinity chromatography on Fast Flow Ni-NTA Sepharose (Qiagen) followed by anion exchange chromatography (MonoQ, GE Healthcare) as described previously [25]. The amount of active enzyme in wild-type InpA preparation was determined by active site titration using inhibitor E64 (Sigma). Briefly, recombinant protein was activated at 37°C for 15 min in 0.1 M Tris-HCl, 5 mM EDTA, pH 7.5 freshly supplemented with 2 mM DTT and then preincubated with increasing concentrations of E64 for 37 min at room temperature. Residual enzyme activity was determined by measurement of fluorescence (λ_ex_ = 380 nm and λ_em_ = 460 nm) of AMC released from Boc-Val-Leu-Lys-AMC (PeptaNova) added to the reaction mixture at 250 µM final concentration and using the microplate spectrofluorimeter SpectraMax Gemini EM (Molecular Devices). The concentration of active InpA was calculated from the amount of inhibitor needed for total inactivation of the proteinase. The final preparations of wild type InpA and InpAC154A were assayed for possible contamination with lipopolysaccharide using Limulus test (Hycult Biotechnology) and found to contain 7 and 1 ng/mL lipopolysaccharide, respectively. Arginine-specific (HRgpA and RgpB) and lysine-specific (Kgp) gingipains were purified from the *P. gingivalis* HG66 strain culture fluid as described previously [5]. Before using in any assay, InpA and InpAC154A were preactivated for 15 min by incubation in a buffer specific for the particular assay supplemented with 2 mM DTT.

### Measurement of InpA activity using fluorogenic substrate

InpA was activated by 15 min incubation in 0.1 M Tris·HCl, pH 7.6, 5 mM EDTA, 2 mM DTT at 37°C. InpA was mixed with increasing concentrations of NHS and incubated for 30 min at 37°C. Control samples without serum and with E64 were prepared simultaneously. After incubation, the substrate Boc-Val-Leu-Lys-AMC was added to all samples, rendering final volume 200 µL and final concentrations of 16.8 nM InpA, 0–30% NHS, 100 µM E64 and 5% DMSO. Substrate hydrolysis was monitored as AMC release. Activity was determined as the initial velocity of the reaction and expressed in relative fluorescence units (RFU)/s. Results from triplicates were plotted using GraphPad Prism software and calculated as relative activity compared to an uninhibited control.

### Bacterial strains and their culture

For detection of *P. intermedia* in clinical samples, subgingival plaque samples were obtained from patients with severe periodontitis (aggressive periodontitis (n = 24), chronic periodontitis (n = 58)). Two paper points were inserted in each pocket for 20 s and DNA was subsequently extracted using the Genomic Mini system (A&A Biotechnology) according to the manufacturer's recommendations. PCR was carried out using primers: Pi-1: TTT GTT GGG GAG TAA AGC GGG and Pi-2: TCA ACA TCT CTG TAT CCT GCG T [44]. Presence of the *inpA* gene was determined using PCR with the following primers that were designed based on Oral Pathogen Sequence Database (gene pPI0032; http://www.oralgen.lanl.gov): pPI-1: GAA GGA CAA CTA CAG CGG AAA; pPI-2: TCC TTT CGT TAG TTC GCT GA. Some of the samples were cultivated on Schaedler agar and Schaedler agar supplemented with 7.5 mg/L vancomycin. Colonies typical for *P. intermedia* were then subcultivated yielding strains 57, 59, 120, 106, BGH10, BGH30, H13 and their identification was confirmed by PCR exactly as described previously [45]. *P. intermedia* OMZ 248 [46], was kindly provided by Dr. Frandsen (Department of Oral Biology, Royal Dental College, Faculty of Health Sciences, University of Aarhus, Denmark). For the experiments conducted in this study, all *P. intermedia* strains were grown on blood-enriched tryptic soy broth (TSB) agar plates at 37°C in an anaerobic chamber (Concept 400, Biotrace) with an atmosphere of 90% N_2_, 5% CO_2_ and 5% H_2_. *Escherichia coli* laboratory strain DH5α (Invitrogen) and *Escherichia coli* clinical strain were grown on standard Luria-Bertani (LB) agar plates or in LB broth. *Prevotella nigrescens* (ATCC 25261) was grown on BBL Columbia II agar containing 8.5% horse blood, 0.04% L-cysteine HCl, 5 mg/mL hemin and 2 mg/mL vitamin K1. Bacterial strains used in this study are listed in Table 2.

**Table 2:** Description of bacterial strains used in this study.

Bacterial strain	Characteristics
*Escherichia coli* DH5α	Common laboratory strain
*Escherichia coli*	Clinical strain isolated from a patient with urinary tract infection.
*P. nigrescens* (ATCC 25261)	Laryngotomy wound
*P. intermedia* (ATCC 25611)	Empyema
*P. intermedia* 59	Severe chronic periodontitis
*P. intermedia* 57	Severe chronic periodontitis
*P. intermedia* OMZ 248	Severe chronic periodontitis
*P. intermedia* 120	Aggressive periodontitis
*P. intermedia* 106	Severe chronic periodontitis
*P. intermedia* BGH 10	Severe chronic periodontitis
*P. intermedia* BGH 30	Severe chronic periodontitis
*P. intermedia* H13	Aggressive periodontitis

### Sampling of crevicular fluid and analysis

Crevicular washes were obtained using a previously described method from 4 patients with chronic periodontitis. For analysis of *P. intermedia* presence, DNA was extracted from 5 µL of crevicular fluid using the High Pure PCR Template Preparation Kit (Roche) according to the manufacturer's recommendations. Real-time PCR was carried out using a RotorGene 2000 (Corbett Research). Primers specific for 16S rDNA from *P. intermedia* were designed as described by [44]. PCR amplification was carried out as described earlier [47]. Determination of InpA in gingival crevicular fluid samples was performed by Western blotting analysis using rabbit polyclonal Ab against 40 kDa (without C-terminal profragment) form of InpA raised in rabbits by standard immunization with purified recombinant InpAC154A.

### Bactericidal assay

Strain *E. coli* DH5α was cultured in LB broth until exponential growth phase. Cells were harvested, washed once in GVB^++^ (5 mM veronal buffer pH 7.3, 140 mM NaCl, 0.1% gelatin, 1 mM MgCl_2_ and 0.15 mM CaCl_2_) and adjusted to an optical density at 600 nm of 0.5. NHS was prepared from blood taken from six healthy volunteers and pooled. NHS was diluted in GVB^++^ to a concentration of 2% and incubated with various concentrations of preactivated InpA or InpAC154A for 15 min at RT. Thereafter, 10^4^ bacteria cells were added and incubated with serum supplemented with InpA for 20 min at 37°C in a total volume of 60 µl. After incubation, aliquots were removed, diluted serially and spread onto LB agar plates. Heat inactivated serum (56°C, 30 min) was used as a negative control. Plates were incubated for 12 h in 37°C after which colonies were counted and percentages of the surviving bacteria were calculated.

*P. intermedia* from four-day old agar plate culture were harvested and washed once in GVB^++^ and adjusted to an optical density at 600 nm of 0.6. Thereafter, 2×10^4^ bacteria were mixed with 20% NHS diluted in GVB^++^ and incubated anaerobically for 1.5 h at 37°C in total volume of 110 µl. The aliquots were removed, diluted serially and spread onto TSB plates. Plates were incubated for 4 days at 37°C in an anaerobic chamber after which colonies were counted and percentages of the surviving bacteria were calculated. *E. coli* were treated in a similar manner except for that 40% NHS was used. All incubations were performed aerobically and the bacteria were spread on LB agar plates for counting colonies after overnight incubation.

### Western blot analysis of interpain A expression

*P. intermedia* strains OMZ 248, 59, 57, 120, 106, BGH 10, BGH 30, H13 and ATCC 25611 were cultured in the Schaedler liquid medium at 37°C in an anaerobic chamber for 5 days. Aliquots of cell culture media adjusted to an optical density at 600 nm of 2.0 were separated under reducing conditions by SDS-PAGE electrophoresis using 12% gel. The proteins were transferred onto PVDF membrane using semi-dry blotting system. After blocking with 50 mM Tris-HCl, 150 mM NaCl, 2 mM CaCl_2_, 0.1% Tween 20 and 3% fish gelatin, pH 8.0, InpA was visualized using an anti-InpA polyclonal antibody (1∶500 dilution) followed by goat anti-rabbit Abs conjugated to HRP and developed using enhanced chemiluminescence (ECL). The signals were collected using CCD camera (LAS3000, Fujifilm).

### Hemolytic assay

To assess activity of the classical pathway, sheep erythrocytes were washed three times with DGVB^++^ buffer (2.5 mM veronal buffer pH 7.3, 70 mM NaCl, 140 mM glucose, 0.1% gelatin, 1 mM MgCl_2_ and 0.15 mM CaCl_2_). The cells were incubated with a complement-fixing antibody (amboceptor; Boehringverke; diluted 1∶3000 in DGVB^++^ buffer) at a concentration of 10^9^ cells/mL for 20 min at 37°C. After two washes with DGVB^++^, 5×10^8^ cells/mL were incubated for 1 h at 37°C with 1.25% NHS diluted in DGVB^++^ buffer (total volume 200 µl). Before incubation with erythrocytes, NHS was pre-incubated with various concentrations of preactivated InpA or InpAC154A for 15 min at RT. The buffer used for activation of InpA did not interfere with the hemolytic assay or erythrocytes (data not shown). The samples were centrifuged and the amount of lysed erythrocytes was determined by spectrophotometric measurement of the amount of released hemoglobin (405 nm).

To assess activity of the alternative pathway, rabbit erythrocytes were washed three times with Mg^++^EGTA buffer (2.5 mM veronal buffer, containing 70 mM NaCl, 140 mM glucose, 0.1% gelatin, 7 mM MgCl_2_, 10 mM EGTA, pH 7.3). Erythrocytes at a concentration of 5×10^8^ cells/mL were then incubated for 1.5 h at 37°C with 10% NHS diluted in Mg^++^ EGTA buffer (total volume 200 µl). NHS used was pre-treated with various concentrations of preactivated InpA or InpAC154A for 15 min at RT. The samples were centrifuged and the amount of lysed erythrocytes was determined spectrophotometrically.

### Complement activation assays

Microtiter plates (Maxisorp; Nunc) were incubated overnight at 4°C with 50 µl of a solution containing 2 µg/mL human aggregated IgG (Immuno), 100 µg/mL mannan (Sigma, M-7504) or 20 µg/mL zymosan (Sigma, Z-4250) in 75 mM sodium carbonate (pH 9.6). Between each step of the procedure, the plates were washed four times with 50 mM Tris-HCl, 150 mM NaCl, and 0.1% Tween 20 (pH 7.5). The wells were blocked with 1% BSA (Sigma) in PBS for 2 h at RT. NHS was diluted in GVB^++^ buffer and used at a concentration of 2% for C3b, C4b, C1q (classical pathway), 4% for C3b, C4b, MBL (lectin pathway), 6% for C3 (alternative pathway) and 10% for C9 (all three pathways). These concentrations were chosen on the basis of initial titrations. NHS was mixed with various concentrations of preactivated InpA or InpAC154A and incubated in the wells of microtiter plates for 45 min at 37°C for C9 and MBL and 20 min at 37°C for C3b and C4b in case of the alternative and the lectin pathways. For the classical pathway, NHS was incubated with preactivated InpA or InpAC154A for 15 min at RT in eppendorf tubes and the enzyme was inhibited by addition of 20 µM E-64 (Calbiochem) to avoid degradation of IgM deposited on plates. Immediately after addition of inhibitor, NHS was incubated in microtiter plates for 45 min at 37°C for C9 and C1q and 20 min at 37°C for C3b and C4b. The inhibitor itself did not affect activation of complement at the concentration used (data not shown). Complement activation was assessed by detecting deposited complement factors using rabbit anti-C1q, anti-C4b, anti-C3d polyclonal antibodies (pAbs, DakoCytomation) goat anti-C9 pAb (Complement Technology) and goat anti-MBL (R&D) diluted in the blocking buffer. Bound antibodies were detected with HRP-labeled anti-rabbit or anti-goat secondary pAb (DakoCytomation). Bound HRP-labelled pAb were detected with 1,2-phenylenediamine dihydrochloride (OPD)-tablets (DakoCytomation) and the absorbance was measured at 490 nm.

To assess deposition of purified C1q on microtiter plates without any complement activator, plates were blocked with 1% BSA in PBS for 2 h at RT. NHS was diluted in GVB^++^ buffer to 4% and mixed with various concentration of interpain A. Plates were incubated for 45 min at 37°C with shaking and the deposited C1q was detected with specific antibodies.

### Deposition of C1q on bacteria

*P. nigrescens* ATCC 25261 from two-day old agar plate cultures were harvested, washed twice in GVB^++^ buffer and adjusted to an optical density at 600 nm of 1.0. NHS was diluted in GVB^++^ to a concentration of 5%, mixed with 6×10^5^ cells and incubated with various concentrations of preactivated InpA or InpAC154A for 30 min at 37°C. Thereafter, the cells were washed twice in the binding buffer (10 mM HEPES, 140 mM NaCl, 5 mM KCl, 1 mM MgCl_2_, 2 mM CaCl_2_, pH 7.2). C1q deposition was assessed by incubation of the cells with rabbit anti-human C1q FITC-conjugated polyclonal antibodies (DakoCytomation, diluted in the binding buffer 1∶100) for 1 h. The cells were washed twice in the binding buffer and finally resuspended in flow cytometry buffer (50 mM HEPES, 100 mM NaCl, 30 mM NaN_3_, 1% BSA; pH 7.4). Flow cytometry analysis was performed using FACS Calibur (Beckton Dickinson).

### Degradation assay

C4 and C3 (0.8 µM each) were incubated with InpA at concentrations ranging from 50 nM to 1250 nM. Incubations were carried out in 0.2 M Tris-HCl, pH 7.4, containing 0.1 M NaCl, 5 mM CaCl_2_ and 2 mM DTT for 30 min at 37°C. For the time course experiment, C4 and C3 (0.8 µM each) were incubated with 640 nM InpA for 5, 10, 20, 30, 45, 60 and 75 min. The proteins were separated by SDS-PAGE electrophoresis using standard Laemmli procedure and 12% gels. Prior to electrophoresis the samples were boiled for 5 min at 95°C in a sample loading buffer containing 25 mM DTT and 4% SDS. After separation, the gels were stained with silver salts to visualize the separated proteins and quantified by densitometry using ImageGauge (FujiFilm, Tokyo, Japan).

### N-terminal sequencing

To determine sites of cleavage by InpA, 10 µg of C3 and C4 were incubated with 500 nM preactivated InpA for 2 h at 37°C and the proteins were separated by 12% SDS-PAGE under reducing condition. The proteins were then transferred to PVDF membranes (Pall) and stained using Coomassie Blue. Bands of interest were excised and analyzed by automated Edman degradation in an Applied Biosystems PROCISE 494 HT sequencer with on-line phenylthiohydantion HPLC analysis using a 140 C Microgradient System from Applied Biosystems, operated according to the manufacturer's recommendations.

### Kinetic measurements using surface plasmon resonance (Biacore)

The analysis was performed according to a previously published protocol [48]. Human C3b was diluted in 10 mM Na-acetate pH 4.0 to a concentration of 30 µg/mL and immobilized on chip CM5 to a level of 3000 RU using amino coupling kit (Biacore) and Biacore 2000. Interpain A was pre-activated by 15 min activation at 37°C in the running buffer (10 mM HEPES, 150 mM NaCl, 1 mM MgCl_2_, 0.15 mM CaCl_2_, 0.005% Tween 20, 0.2 mM DTT; pH 7.4) with 2 mM DTT and diluted in the running buffer in a concentration range 0.25–6 µM. Interpain A was then injected at the flow rate of 5 µl/min at 37°C over the immobilized C3met and its activity was quantified as decrease in RU on the sensorgram and analyzed using Biaevaluation software (Biacore).

### Determination of kinetic parameters of C3 and C4 degradation by InpA

Several concentrations of C3 (1.2–7.2 µM) and C4 (0.2–4.8 µM) diluted in DGVB^++^ were incubated with 110 nM or 40 nM of preactivated InpA, respectively. The incubation time was 4 h and 20 min for C3 and C4, respectively. In parallel, the same concentrations of C3 and C4 were incubated without enzyme. Proteins were separated under reducing conditions by SDS PAGE using 12% gel, stained with Coomassie and the gels were scanned followed by densitometry determination of α-chains of C3 and C4 (ImageGauge). Intensity of α-chain bands in the presence of InpA was compared to corresponding controls and expressed as the amount of substrate remaining. Initial velocity of the reaction at each concentration was calculated as amount of substrate consumed within one second and fitted by nonlinear regression into the Michaelis-Menten equation V = (k_cat_*[E]_t_*[S])/([S]+K_m_) using GraphPad Prism. Values *K_m_* and *k_cat_* were obtained as regression curve parameters. Similar values were obtained from two independent experiments.

### Ethics statement

The ethical board of Lund University has approved collection of sera from healthy human volunteers. The ethical committee of Jena University approved collection of periodontal plaques and crevicular fluid. Informed consent was obtained from patients and the investigation was performed according to principles of the Declaration of Helsinki.

### Statistical analysis

Student's t-test was used to calculate the p values in order to estimate if the observed differences between experimental results were statistically significant.
