# Correction: Interpain A, a Cysteine Proteinase from *Prevotella intermedia*, Inhibits Complement by Degrading Complement Factor C3

**DOI:** 10.1371/annotation/e82f810e-738a-47e5-9295-5a0cc9a0dc6c

**Published:** 2009-03-10

**Authors:** Michal Potempa, Jan Potempa, Tomasz Kantyka, Ky-Anh Nguyen, Katarzyna Wawrzonek, Surya P. Manandhar, Katarzyna Popadiak, Kristian Riesbeck, Sigrun Eick, Anna M. Blom

The sixth author's affiliation was incorrect. Surya P. Manandhar is affiliated with #3: University of Georgia, Department of Biochemistry and Molecular Biology, Athens, Georgia, United States of America.

